# Embodied engagement: fostering caring thinking in a multimodal singing-based learning environment for refugee children

**DOI:** 10.3389/fpsyg.2025.1602855

**Published:** 2025-10-22

**Authors:** Mahsa Mohammadhosseini, Silke Schmid

**Affiliations:** Institute of Music, University of Education, Freiburg, Germany

**Keywords:** music education, embodiment, multimodality, refugee children, caring thinking, singing, resilience, socio-emotional

## Abstract

Various initiatives have implemented music programs aimed at fostering refugee children’s resilience and flourishing. However, there is a lack of studies addressing the design of these educational settings with respect to the crucial role of embodiment. Hence, the challenge remains: What approaches can guide educators working with vulnerable groups? Evidence on the effects of embodied musical practices conveys a potential that resonates with fundamentals of Caring Thinking (CT) that stresses embodied, affective components. This study aims to design a Multimodal Singing-Based Learning Environment (MSLE) fostering CT among primary school-aged refugee children. Utilizing a Design-Based Research (DBR) framework, this study develops and iteratively refines the MSLE. It does so by employing the Mosaic Approach, which integrates a range of child-centered methods. Thus, the data encompass thematic drawings and videographed group activities. Focusing on the bodily dimension of children’s musical engagement, the paper explores bodily indicators of caring thinking among *n* = 6 7- to 10-year-old refugee children. Eight interventional workshop sessions incorporated singing in an Atelier Concept (clay sculpturing or shadow play) with a focus on symbolic expression based on the children’s affections. All sessions were videographed. Thematic analysis is used to develop patterns within the data. Initial findings suggest that integrating singing-based embodied learning practices with CT through nonverbal storytelling positively impacts children’s sense of belonging. Findings also indicate that rhythmic breathing techniques may enhance children’s *regulation* and *relaxation responses*. Singing traditional songs appears to facilitate *social cohesion* among participants from heterogeneous backgrounds. Additionally, group singing is associated with increased *emotional expression* and may attenuate *symptoms of anxiety*. The findings also highlight the necessity for facilitators to employ responsive pedagogical strategies to align interventions with the evolving *developmental trajectories* of the children. By focusing on the bodily dimension of musical engagement, the research contributes to the growing body of literature on how music education can support vulnerable populations.

## 1 Introduction

In a rapidly changing society, a growing body of research acknowledges the role of arts-based interventions in fostering human flourishing, particularly among vulnerable populations (Nijs and Nicolaou, 2021). Increasingly, children’s wellbeing is also recognized as a vital objective in educational settings (Schmid, 2024; Silverman, 2023). In particular, music-based interventions have shown promise in cultivating safe and expressive environments that promote empathy, belonging, and emotional care (Kenny, 2023; de Quadros and Amrein, 2023), especially through shared, relational music-making (Hendricks, 2023). In this study, “intervention” denotes planned support activities for student development, tailored to refugee children. While some cited work is school-based, our project primarily concerns out-of-school settings.

Recent discussions in refugee education research emphasize resilience as a key factor in supporting displaced children, particularly in helping them overcome emotional and social obstacles to learning (Ialuna et al., 2024). In the context of this study, resilience is examined not merely as individual trait but as a dynamic process that can be fostered through structured interventions, particularly those that incorporate music-based practices. The concepts of resilience and wellbeing, while interrelated, represent distinct constructs with unique implications for educational interventions among refugee children. Resilience is defined as an individual’s ability to adapt positively to adversity through psychological flexibility and social support mechanisms (Ialuna et al., 2024). Rather than being an innate trait, resilience is a dynamic process influenced by external factors, including supportive relationships and structured educational settings (Deng et al., 2022). In contrast, wellbeing encompasses a broader spectrum of emotional, psychological, and social stability, reflecting long-term life satisfaction and mental health rather than immediate adaptive responses (Juntunen and Sutela, 2023; see also Schmid, 2024). However, there is a notable lack of studies investigating how embodied singing practices can concretely enhance caring thinking among vulnerable populations such as refugee children Garry and Phelan (2022). While the integration of caring thinking in music-based practices is still emerging, recent arts-based studies have emphasized how singing can act as a participatory and transformative method for supporting inclusion and self-expression in refugee and asylum-seeking communities (Lenette, 2019; Phelan et al., 2025). The concept of caring thinking (Lipman, 2003) emphasizes empathy, ethical reflection, and relational understanding. When integrated with embodied musical activities such as singing, it has the potential to address both emotional and social needs.

The global refugee crisis has created unprecedented challenges for education systems, particularly in addressing the needs of refugee children who face social, emotional, and cognitive disruptions due to displacement and trauma (Kirmayer et al., 2011; Heynen et al., 2022). These challenges often lead to increased anxiety, instability, and difficulty in forming social bonds, emphasizing the need for educational interventions that enhance children’s resilience-helping them develop adaptive coping mechanisms while also promoting overall wellbeing. Arts-based interventions, especially those centered on music, have emerged as powerful tools for fostering resilience, emotional healing, and social cohesion among vulnerable populations (Crawford, 2017; Heynen et al., 2022; Marsh, 2017; Van der Merwe et al., 2023). Within these arts-based interventions, singing stands out as a profoundly embodied activity. Singing integrates controlled breathing, posture, and vocal resonance, activating cognitive, emotional, and physical domains simultaneously (Welch, 2012). It also offers opportunities to foster deeper CT by providing children with a structured environment to explore ethical and emotional dimensions (Lipman, 2003; Sharp, 2017). According to Nicolaou et al. (2023), incorporating movement into musical experiences offers a structured environment where asylum-seeking children can form meaningful social connections and engage in cultural interaction, both of which contribute to resilience-building. Participatory singing activities foster a collaborative environment where children can process complex emotions and develop socio-emotional skills. For example, in asylum-seeker centers, music and movement workshops have proven effective in promoting identity formation, cultural belonging, and emotional expression (Nicolaou et al., 2023; Marsh, 2017).

Embodiment, a concept rooted in cognitive science and education emphasizes the interconnectedness of mind and body in learning. It highlights the role of physical engagement, sensory experience, and affective connection in facilitating deeper understanding and emotional growth (Damasio, 1999; Lewis, 2020; Schmid, 2019). The Dalcroze approach, as explored by Van der Merwe et al. (2023), illustrates how music and movement can contribute to wellbeing by fostering joyful, interactive experiences that promote emotional balance. Additionally, it supports resilience by helping children develop adaptive strategies for navigating social challenges. These activities promote collaboration, mutual respect, and collective creativity, which are essential for enhancing participants’ sense of belonging and resilience in challenging contexts. Participation in musical activities has been shown to promote social integration and a sense of belonging for migrant children. Research suggests that structured music programs can enhance cultural adaptation while fostering peer relationship in educational settings (Rinde and Kenny, 2021). Research by Millar and Warwick (2019) points out that singing also supports language acquisition and social bonding, which are particularly valuable for refugee children facing linguistic and cultural barriers. Additionally, Nicolaou et al. (2023) emphasize the role of artist-facilitators in guiding music-based workshops, using reflexive practice to adapt activities that meet participants’ evolving needs. The integration of these embodied practices with caring thinking (CT) can provide transformative learning experiences for vulnerable groups.

Despite these theoretical insights, there is a lack of systematic research exploring the design and implementation of embodied singing-based interventions to support holistic development in refugee children. The integration of CT into embodied musical practices provides a promising avenue for addressing these gaps. For refugee children, this approach offers a structured way to process emotions, build resilience, and foster a sense of belonging. Singing, as a communal and embodied activity, aligns closely with these principles, enabling children to collaboratively explore emotions and values in a supportive environment (Lewis and Chandley, 2012). Moreover, research demonstrates that embodied practices, such as those inspired by the Dalcroze approach, facilitate meaningful engagement through movement, playfulness, and improvisation (Van der Merwe et al., 2023). Music–movement integration emphasizes the sensory and cognitive interconnectedness that supports emotional wellbeing (Juntunen and Sutela, 2023). Studies have shown that movement-based musical activities can significantly enhance participants’ sense of agency, self-regulation, and social connection, particularly among refugee and other vulnerable populations (Juntunen and Sutela, 2023; Heynen et al., 2022; Crawford, 2017). Such findings reinforce the importance of designing multimodal learning environments that combine singing with movement to promote holistic development. However, there is still a pressing need for systematic studies addressing the concrete design of these educational settings. Educators working with vulnerable groups require principles to design learning environments. To address the limited and fragmented integration of caring thinking within embodied musical practice, this study develops design principles for a Multimodal Singing-Based Learning Environment (MSLE) and examines how embodied participation in singing can foster refugee children’s caring thinking and socio-emotional wellbeing. Adopting a Design-Based Research (DBR) approach, we pilot, test, and iteratively refine the MSLE and analyze children’s embodied engagement using multimodal evidence. The study contributes (i) empirically grounded principles for designing singing-based, embodied interventions and (ii) practice-oriented guidance for educators working with vulnerable groups in out-of-school contexts, alongside (iii) evidence that dialogic, participatory musical spaces can nurture agency and a sense of belonging. Accordingly, we ask the following research questions:

1. What embodied musical practices can be observed in the MSLE which mirror indicators of CT, potentially fostering CT?
2. What implications for educational practice with refugee children can be drawn from these observations?

## 2 Refugee children, embodiment and caring thinking

In the following, we delineate the underpinnings essential for the design of educational settings specifically for vulnerable populations such as refugee children. We commence with an analysis of the psychosocial and educational adversities encountered by refugee populations, emphasizing the imperative for bespoke educational interventions designed to address these challenges. Subsequently, we examine the construct of embodiment and its intersection with musical engagement, highlighting its efficacy in facilitating affective development, before examining the concept of caring thinking as a critical component in supporting social and emotional learning. These elements collectively inform an integrated approach that seeks to empower educators and enhance educational experiences for marginalized groups.

### 2.1 Refugee children

Refugee children face a constellation of adversities, including educational disruption, trauma, social isolation, cultural dissonance, and premature responsibilities within family units (Nicolaou et al., 2023; UNICEF, 2016; Gandham et al., 2023). Prolonged displacement and precarious legal status heighten stress, undermining psychological and social wellbeing (Nielsen et al., 2020). These factors, compounded by limited access to culturally responsive education, increase the risk of long-term socio-emotional difficulties (Heynen et al., 2022; Crawford, 2017).

Arts-based and peer-supported interventions particularly music and movement, offer promising responses. Such practices promote emotional resilience, safe expression, and adaptive coping mechanisms (Van der Merwe et al., 2023). Musical engagement fosters social connection (Heynen et al., 2022), reduces trauma-related symptoms (Crawford, 2017, 2020), and supports emotional regulation (Millar and Warwick, 2019; Juntunen and Sutela, 2023). In addition, music-making enables agency, intercultural belonging, and identity formation (Phelan et al., 2025; Marsh, 2017; Hess and Bradley, 2021).

Empirical studies confirm the impact of these interventions. Dalcroze-inspired workshops in South Africa empowered communities, including children to address shared challenges through embodied musical expression (Van der Merwe et al., 2023). Syrian refugee children reported reduced anxiety and depression following arts-based programs (Ugurlu et al., 2016). Similarly, Nicolaou et al. (2023) identified key pedagogical elements -non-verbal communication, active participation, and non-linear design- across music workshops in asylum centers. Their research, grounded in Communities of Musical Practice (Kenny, 2016), highlights the importance of culturally responsive, participatory approaches principles also endorsed by UNHCR (2024).

Singing, in particular, offers a socially cohesive and embodied practice that can restore agency, belonging, and emotional stability for displaced children (Vougioukalou et al., 2019).

### 2.2 Concepts definition: embodied practices, singing and caring thinking

*Embodied musical practices* hold particular relevance in educational interventions addressing trauma and displacement. As Nicolaou et al. (2023) emphasize, music and movement are deeply interconnected, with bodily engagement playing a central role in singing. Dalcroze Eurhythmics exemplifies this by integrating rhythmic movement with music to enhance emotional regulation, coordination, and social interaction (Juntunen, 2019; Van der Merwe et al., 2023). Juntunen (2019) highlights how such practices activate a “perception–action loop,” linking movement, listening, and cognition into cohesive learning experiences. Similarly, Juntunen and Sutela (2023) show that music, particularly singing, supports emotional modulation, motor control, and interpersonal connection.

*Singing*, as an embodied and inclusive practice, merges sensory, emotional, and cognitive engagement. It functions as “augmented speech” (Ruksenas, 2012), enabling self-expression and emotional processing, particularly for vulnerable groups such as refugee children. As individuals “become the instrument” (DeLorenzo, 2003), barriers to participation are reduced. Singing also fosters collective action and empathy acting “in unison” (Nicolaou et al., 2023), and aligns with Schmid’s (2016) framework that integrates embodiment, narrativity, and sociality in children’s musical experience. Schmid (2024) and Holdhus (2023) view musical flourishing as a form of care, where joyful, active engagement enhances resilience and wellbeing. Renzoni (2018) further underscores singing’s flexibility in navigating diverse cultural contexts through embodied playfulness and cooperation. Beyond its expressive and regulatory functions, singing also serves as a gateway to relational and ethical engagement, forming a conceptual bridge to Caring Thinking.

*Caring Thinking* (CT), conceptualized by Lipman (2003) in Philosophy for Children (P4C), emphasizes ethical reflection, emotional intelligence, and empathy. Defined as “thinking in values,” CT fosters relational awareness between emotion and cognition. Hendricks (2023) extends this to music education, advocating for co-constructed, non-hierarchical learning relationships rooted in shared humaneness. CT aligns closely with social–emotional learning objectives, especially in refugee contexts, by promoting belonging, agency, and compassion (Ventista, 2018; Ab Wahab et al., 2022). P4C-inspired programs use “communities of inquiry” to nurture empathy and critical dialogue, while music education offers unique opportunities to integrate CT through affective, embodied, and collaborative experiences.

## 3 Material and methodology

### 3.1 Design-based research approach

The data of this study was collected in the context of a Design-Based Research (DBR) methodology, which is ideal for developing and refining educational interventions. DBR is an iterative and participatory approach that involves cycles of design, implementation, evaluation, and refinement. This methodology allows for continuous adaptation of interventions based on real-time feedback and emergent data, making it particularly effective for addressing the complex and evolving needs of refugee children (Bakker and Erde, 2023). The DBR approach was framed within a sociocultural learning perspective, which emphasizes the dynamic relationship between individuals and their environments (Ryu, 2020, p. 5). This framework is especially relevant for working with displaced children, as it highlights the embodied nature of learning and acknowledges the role of cultural expression in identity formation (Liebenberg et al., 2020). The flexibility of DBR, along with its emphasis on collaboration, made it an ideal methodology for ensuring the intervention, i.e., the development of the MSLE remained contextually and culturally relevant.^1^

### 3.2 Researcher-as-instrument statement

In line with Morrow’s (2005) recommendations for qualitative research, we provide an overview of our backgrounds as researchers to enhance transparency regarding our perspectives and potential influences on the study. The first author holds a BA in Sociology and has been a facilitator of Reggio Emilia and P4C methodologies since 2010, working with children from diverse backgrounds. Her background in child-centered learning shaped the conceptual foundations of the MSLE (see 3.4). She consulted three experts in qualitative research methodology, P4C, and Caring Thinking to ensure theoretical and methodological rigor. The second author is a scholar in music education with expertise in multimodal learning, embodied cognition, and participatory research. She contributed to methodological oversight, theoretical refinement, and the integration of music-based interventions in the study. To maintain reflexivity, the research incorporated expert feedback, peer review, and iterative analysis cycles, ensuring the credibility and trustworthiness of the findings.

### 3.3 Context: setting, qualitative data collection and analysis

To gain deep insight into the participants´ experiences in the context of the MSLE, a qualitative approach was chosen. A first step identified principles considerate of (a) the target group’s specific needs, (b) multimodal singing-based and (c) caring thinking methods. Thus, prototypical design principles using a hybrid design with games, infographics, images, and storytelling form the basis for the MSLE, establishing of a *community of Inquiry* (Sharp, 2017). In this context, data was collected and analyzed. However, the implementation of the MSLE was not without challenges. Linguistic diversity was a significant obstacle, as children from different cultural and linguistic backgrounds had varying levels of proficiency in the language of instruction. This necessitated reliance on embodied and non-verbal modalities already central to the study’s theoretical framework (see 3.1). Additionally, cultural differences in learning styles and prior educational experiences influenced how children engaged with the activities, necessitating adaptive pedagogical approaches. Another key challenge was the emotional sensitivity of participants, as some children, due to past experiences of displacement and trauma, were initially hesitant to engage. Establishing a sense of trust and psychological safety required sustained effort, and some children needed multiple sessions before feeling comfortable participating in singing, movement, or storytelling exercises. Moreover, logistical constraints, such as limited space, fluctuating attendance, and the unpredictability of life in a refugee camp setting created additional barriers to structuring consistent learning experiences. These challenges align with findings from Warner and Groarke (2022), who highlight how navigating complex systems often imposes additional burdens on vulnerable populations. Similar to how individuals managing hereditary health conditions experience uncertainty in accessing tailored healthcare services, refugee children in educational settings face structural and institutional barriers that affect their ability to engage with learning environments. Addressing these challenges requires flexible, culturally responsive teaching approaches that accommodate the diverse needs, backgrounds, and emotional states of refugee children. Despite these obstacles, the adaptive and participatory nature of the MSLE allowed for context-sensitive instructional modifications. Strategies such as non-verbal storytelling, improvisation, and multimodal engagement helped bridge linguistic and cultural gaps, while the structured yet flexible framework focused the fostering of trust, confidence, and active participation. These observations underscore the importance of integrating trauma-informed, embodied pedagogies when designing educational interventions for refugee populations.

#### 3.3.1 Research setting and participants

The study was conducted at the refugee camp *Kappler Knoten* in Freiburg, Germany, involving temporary accommodation and integration programs for displaced families. The workshops were held in a quiet, empty room to minimize external distractions and allow children to fully engage in the activities. The study involved eight weekly workshops over 1 month, held twice a week, conducted during the summer of 2024, each lasting 1 h. The primary participants were six refugee children (3 boys, 3 girls), aged 7 to 12 years. Additional children participated occasionally based on availability and interest. The participants came from Kurdistan, Serbia, Syria, and Ukraine, representing diverse linguistic and cultural backgrounds. Hence, the sampling reflected the transient nature of refugee communities (Woodard, 2024), allowing for a dynamic and flexible group composition. A purposive sampling strategy was applied to maintain a balanced and inclusive group composition, ensuring representation of different experiences among refugee children. The facilitator, familiar with the children’s sociocultural contexts, conducted the research as a participant-observer, designing and leading activities, facilitating engagement, and documenting responses. This approach ensured that the children’s experiences were carefully observed and interpreted, integrating both educational and emotional dimensions (Clark and Moss, 2005). The workshop framework draws conceptual inspiration from *White Ant Atelier*, an international online atelier founded by M. Mohammadhosseini in response to the COVID-19 pandemic. It integrates Reggio Emilia and Philosophy for Children (P4C) methodologies to foster creative and reflective learning experiences (Table 1). ^2^

**Figure 1:**
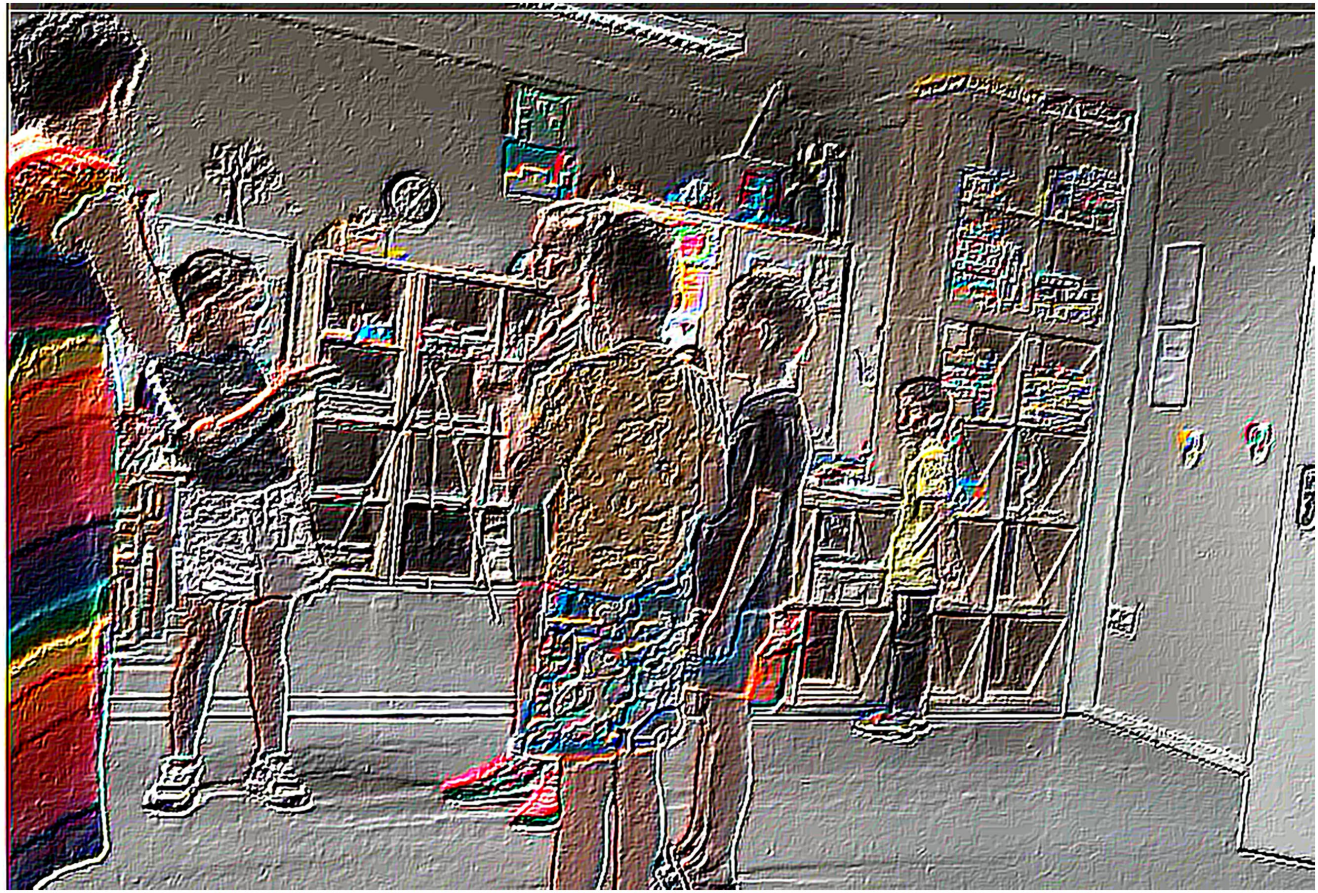
Children mirroring and exaggerating poses during the storytelling session, illustrating playful imitation and emotional connection.

**Table 1:** Overview of workshop themes and participation numbers.

Workshop	Focus area	Learning experience	Engagement style	Number of children
Rhythmic motion and storytelling	Exploring personal expression through movement and music	Participants engage in activities where they express ideas and stories using coordinated body percussion and guided movement improvisation. The workshop integrates rhythm with narrative development to enhance creative storytelling skills.	Interactive exercises, group collaboration, and guided improvisation	7–11
Visualizing sound through motion	Connecting bodily gestures with auditory and visual elements	Through the use of colored fabric and expressive motion, children learn to interpret sounds through movement. They also explore how emotions influence gestures and how these can be transformed into visual representations.	Role-playing, symbolic movement, and group discussion	6–10
Melodic exploration and philosophical inquiry	Combining vocal practice with instrumental play and deep thinking	Participants experiment with different melodic structures using color-coded instruments and group vocal exercises. They also reflect abstract ideas and ethical themes, integrating them into collaborative songwriting activities.	Structured singing, reflective dialogue, and group composition	5–8
Music, emotion and personal reflection	Using rhythm and melody to explore emotions and values	Children explore the relationship between mood and rhythm through movement-based activities. They create and perform original compositions that reflect their personal experiences, followed by artistic discussions and reflections.	Expressive performance, improvisation, and discussion-based engagement	8–11

#### 3.3.2 Data collection methods

The data collection process employed a Mosaic Approach, a participatory research methodology designed to capture children’s diverse experiences through multimodal techniques. The approach emphasized children’s perspectives and co-construction of knowledge, aligning with the study’s sociocultural and embodied learning framework (Clark, 2005; Ryu, 2020). This method was chosen because of its flexibility in capturing non-verbal communication and children’s agency in the research process (Nicolaou et al., 2023). It allowed for a holistic representation of children’s embodied musical engagement, ensuring that their experiences were rendered through multiple data sources. To analyze non-verbal communication, video recordings were crucial to capture gestures, facial expressions, and movement-based interactions. Importantly, children were actively involved in the setup and dismantling of the cameras, fostering a sense of agency and participation in the research process. This involvement not only helped them become familiar with the documentation tools but also ensured a more natural and engaging environment during recordings. The data collection integrated structured observations, video recordings, verbal reflections, and embodied responses, ensuring a comprehensive and ethically responsible method of documenting children’s experiences (Clark and Moss, 2005). The recordings were complemented by facilitator notes, thematic reflections, and participant observations, which were systematically categorized using thematic and phenomenological analysis techniques (Nicolaou et al., 2023).

The video cameras were placed at fixed angles to ensure minimal disruption while capturing holistic body movements essential to the study’s focus on embodied musical engagement. Children were also given the opportunity to express their experiences through drawings, storytelling, and participatory feedback sessions, reinforcing the inclusive and participatory nature of the study. These structured yet flexible documentation strategies ensured that children’s voices and emotions were authentically represented, contributing to a rich, multidimensional dataset (Clark and Moss, 2005; Nicolaou et al., 2023).

#### 3.3.3 Data analysis procedures

The collected data were analyzed employing an adaptation of thematic analysis (TA), following Braun and Clarke’s (2006) structured framework. TA was chosen because of its flexibility in capturing both explicit and implicit meanings in qualitative data, making it particularly suitable for analyzing embodied experiences and non-verbal interactions (Braun and Clarke, 2023). Unlike other qualitative analysis methods, thematic analysis allows for an iterative process, enabling a balance between researcher-led coding and participant-driven insights (Liebenberg et al., 2020). This was crucial in ensuring that children’s embodied expressions were analyzed in a structured yet interpretative manner, aligning with the study’s focus on social connection and emotional engagement through movement. Using the MAXQDA, the analysis followed six key stages: (1) Familiarization with the data – reviewing video recordings, observational notes, and facilitator diaries to develop an in-depth understanding; (2) Generating initial codes – identifying meaningful features and recurring patterns in the dataset; (3) Identifying key themes – clustering codes into broader themes that represented children’s experiences; (4) Reviewing and refining themes – ensuring that the themes accurately reflected the dataset and aligned with embodied learning frameworks; (5) Defining and naming themes – finalizing thematic categories based on conceptual coherence; and (6) Producing the final report – synthesizing findings to answer the research questions.

To enhance methodological plasticity and in line with the mosaic strategy, a triangulation approach was employed, integrating three complementary data sources as “individual tiles” (1) Video data, capturing children’s embodied engagement, gestures, and non-verbal expressions, providing insights beyond verbal discourse. (2) Field notes, systematically documented after each session in accordance with P4C and Reggio Emilia documentation principles. These notes followed a structured format, encompassing (a) detailed observations of children’s interactions and responses, (b) facilitator reflections on pedagogical approaches, and (c) potential modifications for subsequent sessions. Session transcripts, capturing verbal interactions and the overall progression of discussions and activities to ensure a comprehensive textual representation of the learning environment. This triangulation methodology facilitated cross-validation between data sources, thereby enhancing interpretative depth and analytical reliability (Erickson, 2011). Furthermore, ethnomethodological videographic analysis was applied to examine bodily expressions, facial cues, and movement-based interactions, offering a nuanced understanding of non-verbal modes of communication (Bühler-Niederberger, 2020). Given the multimodal nature of the data, particular attention was devoted to the transcription process. A total of eight video was reviewed three times by researcher 1 to meticulously document both verbal and non-verbal cues, thereby ensuring interpretative thoroughness. All video data were included in the thematic and multimodal analysis, ensuring comprehensive coverage of children’s embodied engagement across the intervention. The video data were analyzed using a multimodal, interpretive framework grounded in sociocultural learning theory [Vygotsky, as discussed in Mahn (2012) and supported by established practices in multimodal transcription and analysis Bezemer and Mavers (2011)]. Drawing on these foundations, the analysis considered gestures, gaze, spatial orientation, and rhythm as meaning-making tools that reflect children’s embodied engagement and social interaction. To ensure analytical depth, video segments were transcribed multimodally, incorporating not only speech but also body movements, gaze direction, and affective expressions, following principles for multimodal transcription (Bezemer and Mavers, 2011; Cowan, 2018). Although no automatic emotion recognition software was used, the interpretation of non-verbal affective states (e.g., expressions of joy, frustration, or collaboration) was inspired by interdisciplinary approaches to multimodal emotion representation (Mocanu et al., 2023).

To enhance the richness of the analytical process, external expert consultation was sought not only for general validation but also for specific aspects of the coding process. Two researchers specializing in Caring Thinking (CT) and Philosophy for Children (P4C) were consulted to examine the alignment between observed behaviors and Lipman’s CT dimensions (Lipman, 2003). Their feedback focused particularly on the interpretation of non-verbal, embodied expressions such as empathic gestures, spatial proximity, and collaborative movement, which were treated as indicators of relational reasoning and emotional attentiveness. These expert insights contributed to refining the operationalization of CT in a multimodal, embodied context. Additionally, preliminary findings were discussed with peers and a co-researcher to ensure analytical transparency and critical scrutiny. The analysis was subjected to three iterative review.

### 3.4 Design and implementation of the learning environment

#### 3.4.1 Description of the multimodal singing-based learning environment

In response to the challenges outlined in Section 3.3, the MSLE design emphasized non-verbal and embodied strategies. Consistent with Nicolaou et al. (2023), selected activities required minimal verbal interaction and supported symbolic expression through voice, gesture, and movement.

Specific multimodal strategies were incorporated, engaging the children through auditory, visual, and kinesthetic methods (Hogle, 2020), such as singing without lyrics, a specific mode of nonverbal group conversations. Concurrently, and in line with existing research (see 2.2), the sessions were designed based on principles of Dalcroze Eurhythmics, the Reggio Emilia approach, and other established methods of community music (e.g., body percussion), empowering the children in symbolic expression (Arseven, 2014; Kenny, 2022a; Kenny, 2022b), empowering children through embodied and symbolic forms of expression (Juntunen and Hyvönen, 2004).

Multimodality and participatory engagement, as core concepts of the MSLE, involved a variety of materials and methods. This aligns with the second and third principles emphasized by Nicolaou et al. (2023), namely, *active participation* and a *non-linear approach* (p. 9). Hence, the workshop sessions centered around vocal emotion, body percussion and vocal storytelling. Tasks like developing and presenting nonverbal vocal tales, which focus on illustrating narrative progressions and emotional experiences, aim to enhance children’s skills such as emotional modulation. Each session included three core components:

1. Singing and Vocal Expression: Culturally relevant songs and improvisational vocal activities to build confidence and promote group cohesion (Kenny, 2022a; Kenny, 2022b).
2. Digital Storytelling Tools: A video^3^ composed of still images from a picture book, accompanied by background music, was used to support emotional and narrative engagement. While not animated in the traditional sense, the musicalized visual sequence provided an accessible and non-verbal storytelling stimulus.
3. Embodied Movement: Activities like clapping, dancing, and mimicking animal sounds to integrate music and physical expression (Kenny, 2022a; Kenny, 2022b).

Thus, the integration of embodiment into the design was grounded in embodied music cognition (Nijs and Nicolaou, 2021), emphasizing the interconnectedness of cognition, emotion, and physical movement through musical and bodily interaction. This approach was operationalized through (1) physical engagement in music, where rhythmic activities helped children internalize musical structures through movement (2) culturally relevant vocal improvisation, which allowed children to express emotions through song (Mabus, 2023a; Mabus, 2023b); (3) and multimodal interaction, combining digital storytelling with physical expression to enhance both cognitive and emotional engagement (Woodard, 2024). These methods supported inclusion and resilience-building, aiming at fostering emotional and social wellbeing through caring thinking among refugee children (Crawford, 2017).

#### 3.4.2 Iterative design process

Aligned with the iterative DBR framework (see Section 3.1), the MSLE design was refined through four development phases. The iterative design process unfolded across 4 weeks of bi-weekly workshops, with refinements introduced after each cycle of observation, reflection, and thematic coding. In Week 1, exploratory prototyping revealed challenges with verbal participation due to linguistic diversity; in response, Week 2 sessions integrated more body-based and non-verbal storytelling activities, including gesture-driven vocal play and collective movement. Ethnomethodological observations in Week 2 highlighted social asymmetries during performance planning, prompting the introduction of structured turn-taking protocols and leadership rotation in Week 3 (e.g., Workshop 5, Transcript Section 9). Thematic refinement took place continuously through facilitator reflection and real-time feedback; for example, following a resource conflict in Workshop 3, collaborative decision-making tasks were adjusted to include fairness prompts and more group discussion time (see Transcript Section 12). Finally, in Week 4, appreciative thinking activities were adapted to emphasize collective celebration and recognition, as seen in expanded use of group applause, peer feedback, and reflective dialogues (Workshop 7, Transcript Section 18). These adaptive modifications were grounded in ongoing analysis using MAXQDA and guided by Lipman’s Caring Thinking framework, ensuring that the learning environment evolved responsively in both form and content (Liebenberg et al., 2020).

The research was conducted in accordance with ethical^4^ guidelines. Distinctly, the research must be trauma-sensitive, reflecting on power relations and prioritizing fully informed consent, especially given the vulnerabilities of the population involved. All procedures are designed to minimize the risk of distress or re-traumatization, while participatory approaches and ongoing researcher reflexivity address and attempt to mitigate power imbalances throughout the project. Informed consent is treated as an ongoing, interactive process, with information presented in an accessible manner and participants reminded of their rights at all stages. Overall, the research is grounded in equity-based learning and benefits from a researcher with close proximity to the issues, ensuring ethical engagement and reciprocity remains central to the study design. Comprehensive ethical oversight, including community consultation and institutional review, further safeguards participants’ wellbeing. Prior to the study, a background clearance letter from the municipality was obtained. Additionally, an agreement with the camp authorities was signed, ensuring that names, images, and videos of the children involved in the study would not be shared outside the research context.

## 4 Results and discussion

### 4.1 Themes and patterns related to embodiment and development of caring thinking

The thematic analysis conducted for this study followed the guidelines established by Braun and Clarke (2006) to systematically develop themes within the data, focusing on the video transcripts. Through iterative coding, six primary themes emerged, each highlighting how embodied learning activities were attributed as indicators of caring thinking among refugee children. These indicators are grounded in Lipman’s (2003) theory of Caring Thinking, which integrates ethical reflection, emotional engagement, and social responsibility into cognitive processes. Lipman categorizes caring thinking into five key dimensions: Appreciative (valuing and respecting others, e.g., becoming manifest in preserving or praising others), Active (participation and initiative, e.g., becoming manifest in organizing or performing), Normative (ethical awareness and fairness, e.g., becoming manifest in requiring rules or obliging to them), Affective (emotional involvement and support, e.g., becoming manifest in reconciling or encouraging others), and Empathic (compassion and mindfulness, e.g., becoming manifest in considerate of others or imaginative when it comes to their needs). These themes can thus become manifest in verbal and non-verbal interactions, emphasizing the role of physicality, emotional expression, and social interaction.

Using this framework as a heuristic for coding sensitization, the analysis process followed an integrative scheme conducive to the specific research interest in a participatory frame yet informed by the theory of caring thinking. Beginning with an inductive analysis, we then used the theoretically sound indicators of caring thinking (see above) to detect patterns that potentially align with them, effectively combining deductive strategies, which is possible in thematic analysis (Braun and Clarke, 2019, p. 592). Thereby six themes emerged as crucial manifestations of embodied engagement and indicating potential fostering of caring thinking, whereby the indicators run through all six topics, so in each theme, after an example from the data and an explication, a respective link to CT is presented. The themes are (1) Physical expression as medium of emotional engagement, (2) Cultural identity through embodied interaction, (3) Body–Mind Integration Through Emotional Regulation, (4) Playfulness and improvisation as catalysts for caring thinking, (5) Social connection in a safe space, and (6) Cognitive and emotional resilience through embodied learning.

All six themes are related yet shown to display distinctive qualities of interaction that were initiated through the specific parts of the workshop design.

#### 4.1.1 Theme 1: physical expression as a medium of emotional engagement

Embodied actions were a primary means for children to express emotions, often serving as an outlet before verbal articulation.

Example:

*Such interactions occurred,* e.g.*, during the storytelling session about a bird’s migration, where the imitation of flying motions led to group laughter and mimicry of own experienced migration. In one incident, for instance, the children moved freely around the room accompanied by rhythmic drumming, and then froze, holding their body positions like statues. Then, they were encouraged to observe and imitate someone else’s pose, embodying that emotion. During this activity, P4 and P5 mirrored each other’s movements* (see Figure 1)*, laughing as they exaggerated poses (Session 6). The playful imitation apparently fostered the expression of emotional connection and joy, helping them socially engage with the narrative*.

Movements such as clapping, swaying, and often mimicking animals showed facilitation of emotional bonding gestures and expression of group cohesion. The theme tackles interactions that enabled the children to process emotions collaboratively (Sangiorgio, 2018). Thus, several incidents in the data were coded, where the children bodily expressed their emotion inspired by a story told during the session.

The theme comprises two observations:

- Empathy Development through Imitation: Activities like rhythmic drumming provided opportunities for children to mirror each other’s movements, fostering emotional attunement and group unity (Maguire and Delahunt, 2017). Post-activity reflections revealed children comforting peers and recognizing their emotions, demonstrating enhanced empathy (Mahn, 2012).
- Caring Thinking Link: Emotional connection through embodied expression created an environment conducive to empathy and reflective thinking as critical components of caring thinking (Mahn, 2012).

Theme 1 illustrates how embodied interactions facilitated emotional attunement and group cohesion, supporting the development of emotional empathy. Beyond serving as a means of immediate emotional attunement, embodied activities also created a bridge between cognitive learning and emotional exploration. Animated storytelling paired with physical reenactments allowed children to engage non-verbally, facilitating participation despite linguistic barriers. This integration of movement, sound, and collective participation further reinforced emotional connection and empathy-building (Cowan, 2018; Mahn, 2012).

#### 4.1.2 Theme 2: cultural expression through embodied interaction

Example:

*In one activity, P4 shared a traditional clapping game from their country of origin. They demonstrated the rhythmic pattern and explained its significance, which sparked curiosity among peers. The game was then enthusiastically adopted by the group, with children experimenting with* var*iations and integrating their own cultural elements into the play.*

The workshops created a space for children to explore their cultural expression through movement and storytelling, promoting inclusivity and mutual respect.

This exchange highlighted how embodied practices foster cultural dialogue (Cowan, 2018).

The theme comprises two observations:

- Cultural expression and Multimodality: The example referred to the multimodal impact, i.e., the integration of visual and kinesthetic elements, such as storytelling accompanied by physical reenactments, enhanced engagement and cross-cultural understanding (Mahn, 2012).
- Caring Thinking Link: Theme 2 is significant with respect to caring thinking as these interactions facilitated emotional belonging, which is vital for children navigating displacement (Sutoris, 2021).

#### 4.1.3 Theme 3: emotional regulation through body–mind integration

Example:

*During a rhythmic jumping activity, P3 initially stood apart but gradually joined the group, synchronizing her movements with others (T.6). The structured rhythm facilitated emotional regulation, helping her transition from isolation to group participation, illustrating how rhythmic movement contributed to emotional self-regulation and social integration.*

In this theme, interactions demonstrated the role of physical movement during the workshop and how it may have helped children manage emotions, showing self-regulation strategies.

Rhythmic breathing exercises and group activities were used at the start of sessions, resulting in visible relaxation and increased focus among participants (Maguire and Delahunt, 2017).

The theme comprises two observations:

- Emotional Regulation and Trauma Processing: Children’s erratic movements became more synchronized during activities, reflecting their ability to process stress in a structured environment (Denzin, 2020).
- Caring Thinking Link: These practices cultivated mindfulness and emotional awareness, enabling children to engage empathetically in collaborative tasks. Research suggests that mindfulness enhances emotional regulation, non-judgmental awareness, and compassionate responsiveness, all of which are closely linked to the development of Caring Thinking (DeMauro et al., 2019).

**Figure 2:**
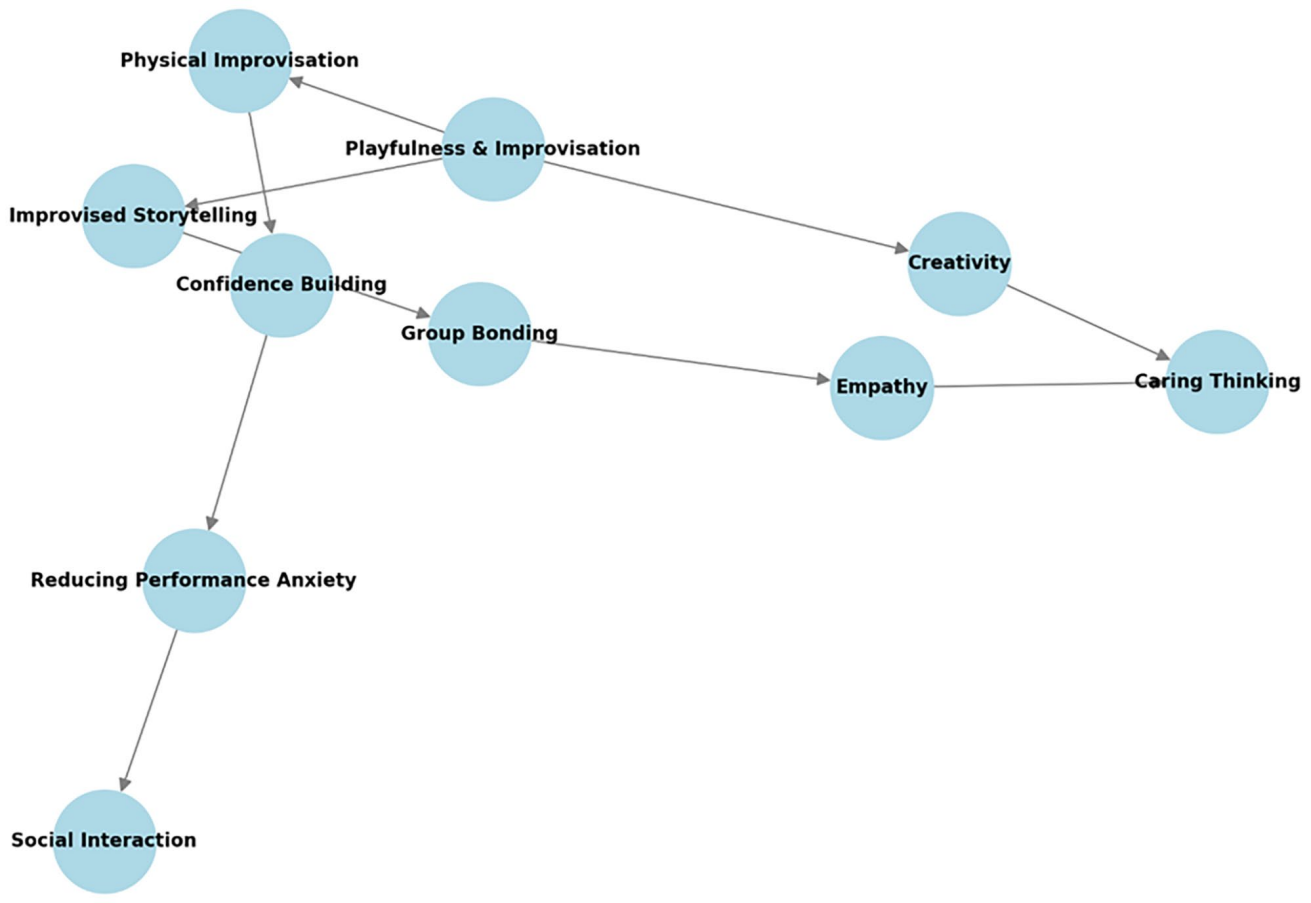
Conceptual network of playfulness and improvisation in caring thinking.

Figure 2 presents a conceptual graph illustrating the interconnections between body–mind integration, emotional regulation, and the development of caring thinking. The relationships depicted in this network highlight how the data analysis brought to light how physical activities such as rhythmic breathing and synchronized movement contributed to practices that displayed practices of mindfulness, stress regulation, and empathy-building among refugee children. This visualization underscores the role of embodied practices in fostering emotional resilience and cooperative learning in theme 3.

**Figure 3:**
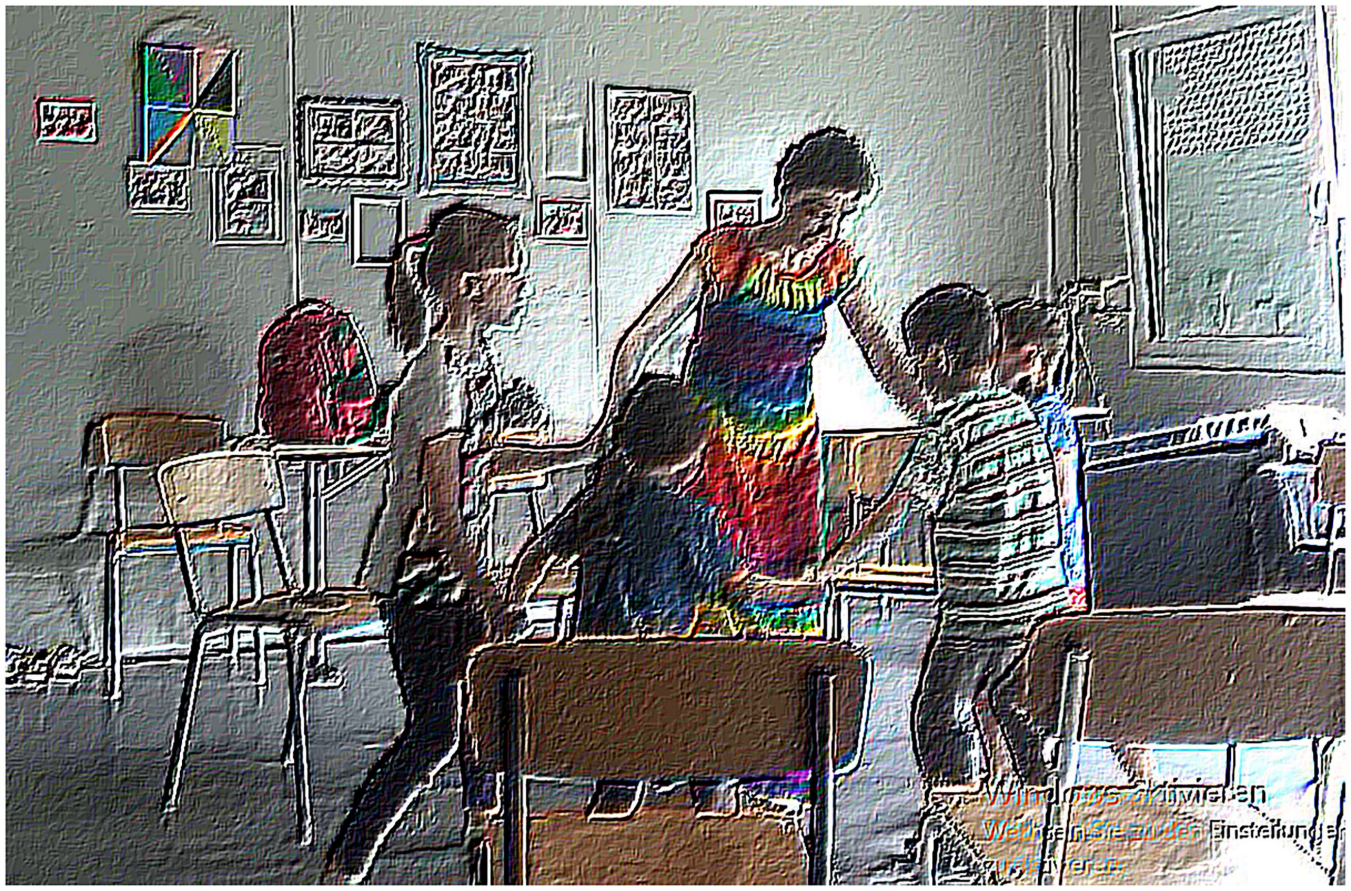
Children holding hands during the playful transition, illustrating enhanced empathy and cooperation.

#### 4.1.4 Theme 4: playfulness and improvisation as catalysts for caring thinking

Playful and improvisational activities encouraged creativity, collaboration, and empathy.

Example:

*In one interaction, P1 and P2 playfully wrestled but then musically transitioned into holding hands and running together in the centre of the circle (T.2). This playful, improvisational transition encouraged visible bonding, where all the children, including those initially engaged in conflicting and distracting interactions, held hands* (see Figure 3)*, illustrating how physical play enhanced empathy and cooperative engagement.*

Improvised storytelling allowed children to use sound and motion to create collective narratives, resulting in spontaneous laughter and group bonding (Sangiorgio, 2018).

The theme comprises two observations:

- Playfulness and Confidence Building: Initially hesitant participants gained confidence through repeated opportunities for playful, physical improvisation, such as mimicking animals or leading group activities (Maguire and Delahunt, 2017).
- Caring Thinking Link: Engaging in improvisational play supported social connectedness, perspective-taking, and cooperative problem-solving. Research indicates that playful interactions enhance emotional engagement, mutual understanding, and prosocial behavior, all of which contribute to the development of Caring Thinking (DeMauro et al., 2019).

**Figure 4:**
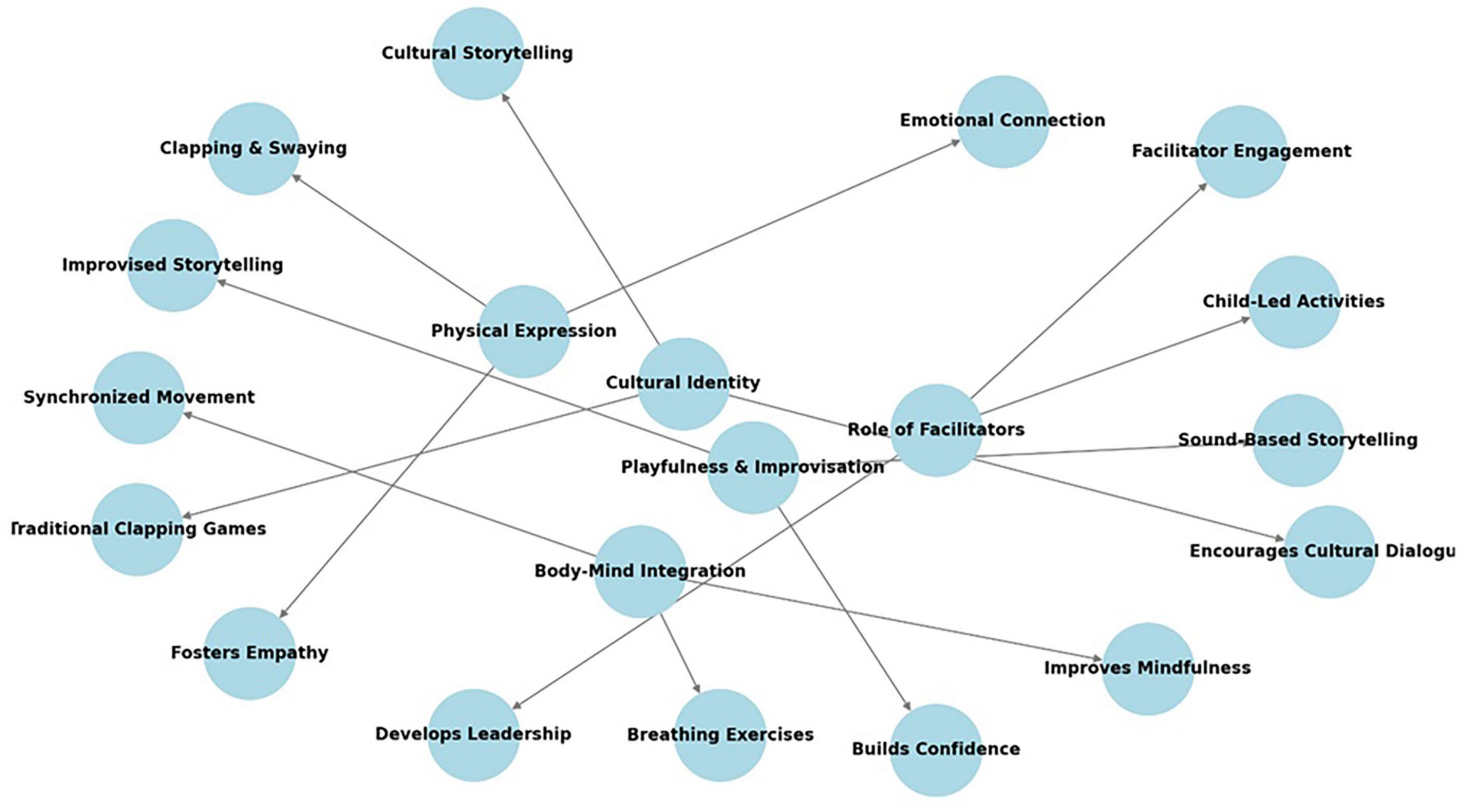
Conceptual network of themes and their impact in embodied learning.

Figure 4 presents a conceptual network illustrating the relationships between the identified themes, key elements, their impact on children, and practical examples from the study. Each theme (e.g., Physical Expression, Cultural Identity, Body–Mind Integration) is connected to its corresponding key elements (e.g., Emotional Connection, Cultural Storytelling), demonstrating how the data analysis revealed emergent traces of caring thinking especially in moments of playfulness and improvisatory elements. The visual representation highlights the interplay between these factors, emphasizing their collective role in promoting emotional resilience, social interaction, and cognitive development among refugee children.

#### 4.1.5 Theme 5: social connection in a safe space

Obviously, the workshops also offered opportunities to foster trust and belonging.

Example 1: *Reduction in isolation through interactive play: In one interaction, During the free play session, P4 and P6 initially stood at the edges, observing but not engaging. The facilitator, maintaining a relaxed, open posture, subtly mirrored their small movements to encourage participation. As the group clapped rhythmically, P6 began tapping their fingers on their knee, gradually syncing with the beat. The facilitator acknowledged this with a gentle nod and synchronized tapping, reinforcing the connection. Meanwhile, P4 took hesitant steps toward the group, clapping softly in response to the facilitator’s cues. Gradually, both children moved closer, their posture becoming more open and engaged. They made eye contact, stepped into the circle, and synchronized their claps with the group’s rhythm. Their transition from passive observation to active participation highlights how embodied musical engagement fosters social inclusion (T.4). The flexible environment obviously encouraged withdrawn children to transition from isolation to active social engagement*.

Example 2: *Peer Support in Group Cohesion: In one interaction, P2 excitedly clapped his hands while requesting the ball from the facilitator. After receiving it, other children spontaneously joined in clapping for him, reinforcing a shared sense of encouragement (T.3). This supportive moment reinforced group cohesion, strengthening social bonds and promoting active participation in collaborative play.*

The safe space apparently allowed children to open up and form meaningful connections across cultural and linguistic divides.

This theme comprises two distinct observations, illustrating different ways in which a safe space fosters social connection. By enabling children to engage in cooperative play and develop trust in their peers, these interactions create a foundation for caring thinking, where empathy and mutual support become integral to their social experience. The first, Reduction in Isolation, reflects how previously withdrawn children gradually transitioned into active participation, underscoring the role of a supportive environment in encouraging social integration. The second, Group Cohesion, captures how collaborative activities, such as rhythmic games, gradually facilitated collective engagement and paved the way to establishing interpersonal bonds.

The theme comprises two observations:

- Group Cohesion: Cooperative activities like rhythmic games generated corporeal indicators for acting out and strengthening interpersonal bonds, with participants showing attentiveness in sharing culturally specific movements and songs (Sutoris, 2021).
- Reduction in Isolation: Facilitator observed that over time, withdrawn children gradually became active participants, highlighting the potential of a safe, supportive environment (Denzin, 2020).

#### 4.1.6 Theme 6: cognitive and emotional resilience through embodied learning

While Theme 3 addresses the immediate role of movement in emotional self-regulation, Theme 6 explores the long-term impact of embodied learning in fostering cognitive and emotional resilience, emphasizing how children independently apply these strategies beyond the workshop environment. The workshops´ features apparently equipped children with tools to regulate emotions and navigate stress, thus potentially fostering cognitive and emotional resilience.

Example:

*During a rhythmic breathing exercise, the facilitator guided the children to inhale deeply and exhale slowly while synchronizing arm movements to a steady beat. This practice was followed by a session of synchronized drumming, where each child imitated the facilitator’s drumming pattern using body percussion. In one instance, P1, who was previously agitated and restless, gradually calmed down as he matched his breathing and drumming to the group rhythm (Session 6). His body movements became more fluid and synchronized, and he visibly relaxed, maintaining focus throughout the rest of the session. The facilitator later observed P1 employing the rhythmic breathing technique independently during a stressful moment outside the workshop, demonstrating emotional self-regulation and cognitive resilience.*

This example illustrates how embodied practices can effectively equip children with emotional regulation tools, supporting long-term stress management and emotional resilience. The theme comprises two distinct observations, each highlighting a different dimension of how embodied learning fosters cognitive and emotional resilience:

- Emotional Regulation: Children independently applied techniques such as rhythmic breathing and synchronized drumming outside the workshop setting to manage stress and anxiety (Maguire and Delahunt, 2017).
- Classroom Impact: The embodied strategies contributed to sustained improvements in focus, collaboration, and emotional engagement in formal learning environments (Sangiorgio, 2018).

**Figure 5:**
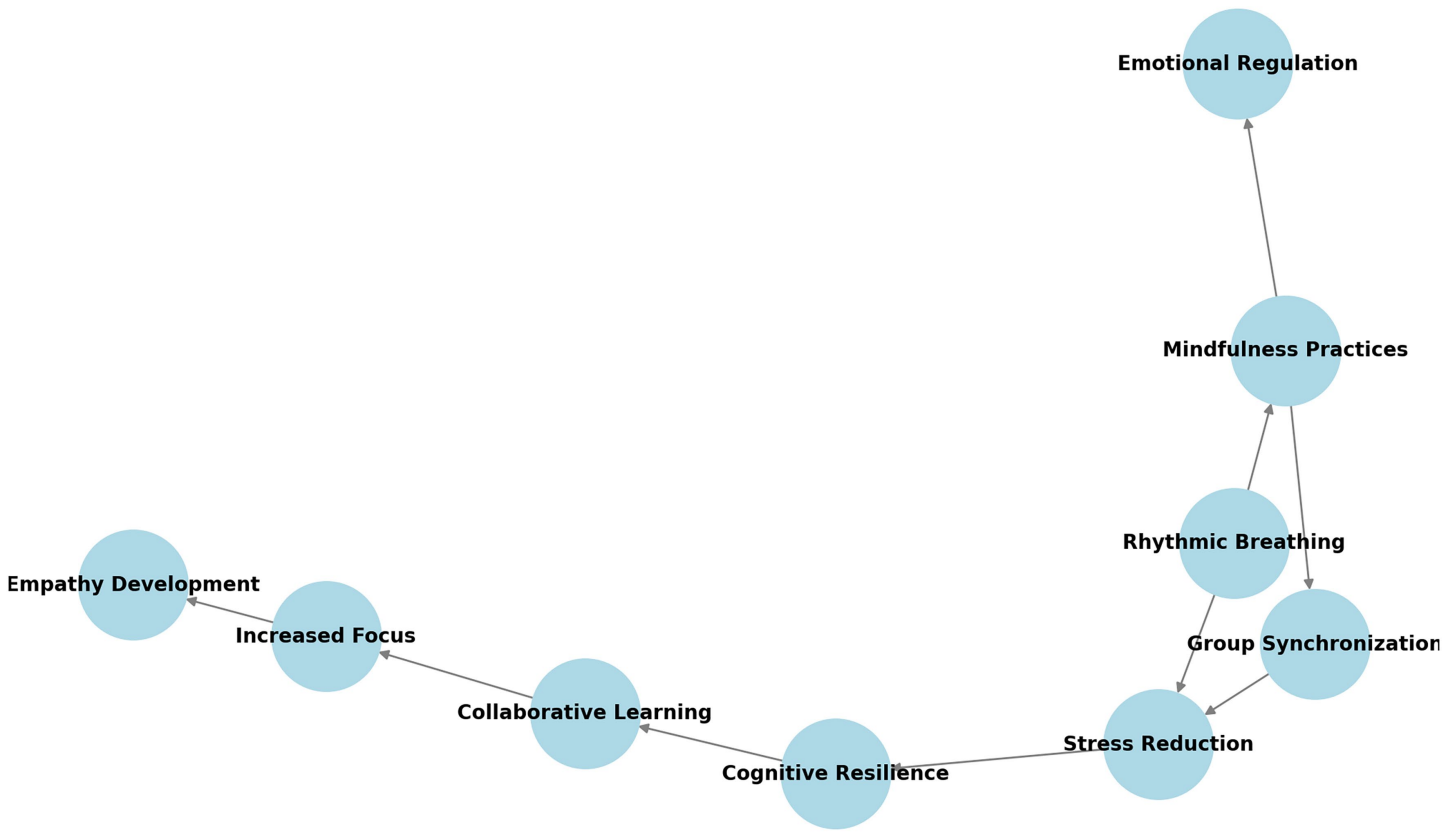
Conceptual graph of emotional regulation and cognitive resilience.

Figure 5 presents a conceptual network illustrating the relationship between emotional regulation and cognitive resilience in the context of embodied learning. The graph highlights how the data analysis indicated that mindfulness practices, such as rhythmic breathing and group synchronization, contribute to stress reduction, which in turn fosters cognitive resilience. Additionally, collaborative learning and increased focus play a significant role in developing empathy among refugee children. This interconnected framework underscores the long-term benefits of embodied learning strategies in supporting emotional wellbeing and adaptive coping mechanisms.

## 5 Implications

### 5.1 Alignment of key findings

This section presents key findings derived from thematic analysis, addressing the study’s research questions. Specifically, it explores (1) the design principles underpinning the development of the MSLE and (2) the impact of embodied musical engagement on caring thinking and socio-emotional wellbeing among refugee children. The six themes identified in this study illustrate how embodied learning practices foster engagement, social connectedness, and cognitive resilience in displaced children. These findings demonstrate how multimodal singing activities, as embodied practices, can be a means to focus on fostering caring thinking among refugee children. The analysis underscores the significance of embodiment in promoting caring thinking and identifies specific types of embodied practices that manifest caring thinking among the participating refugee children within the multimodal learning environment. The six themes which were developed in this research substantiate existing claims concerning the crucial role of embodiment for the design of educational settings. They also conform to seminal embodiment theories: Theme (1) (*Physical Expression as a Medium of Emotional Engagement) and* (2) (*Cultural Identity Through Embodied Interaction*) align with existing research on the benefits of multimodal learning for vulnerable populations (Mahn, 2012; Sangiorgio, 2018) and also resonate with Merleau-Ponty’s (2002) notion of the “lived body,” which views physical interaction as a central part of meaning-making. Theme (3) (*Body–Mind Integration and Emotional Regulation*) and (4) (*Playfulness and Improvisation as Catalysts for Caring Thinking*) illuminate how singing, when combined with controlled breathing, posture, and vocal resonance, creates a dynamic environment where children engage collaboratively, enabling both emotional expression and social bonding. The way group singing sessions fostered synchronized actions and vocalizations in the MSLE apparently allowed children to incorporate a sense of connectedness and safety. Additionally, the role of playfulness in Theme 4 supports Schmid’s (2024, p. 12) assertion how a design centered on playfulness can create assemblages that facilitate a playful understanding between human beings (Arculus, 2020, p. 62). Theme (5) *Social Connection in a Safe Space* underscores the role of a structured and emotionally supportive environment in fostering trust, belonging, and group cohesion. It highlights how collaborative singing and movement-based activities helped children navigate social interactions with confidence, reinforcing peer relationships and interpersonal trust (Hendricks et al., 2014). Theme (6) *Cognitive and Emotional Resilience Through Embodied Learning* renders the potential for a long-term impact of embodied musical practices in enhancing emotional regulation, stress management, and cognitive resilience. Techniques such as rhythmic breathing and synchronized drumming obviously equipped children with adaptive coping mechanisms that extended beyond the workshop setting, fostering sustained improvements in focus and emotional wellbeing. These findings align with prior research on multimodal learning for refugee children. Nicolaou et al. (2023) emphasize that embodied engagement and non-verbal communication foster safe spaces for social bonding, a pattern observed in this study. Similarly, Crawford (2017) highlights how participatory singing supports identity formation and resilience in asylum-seeker communities. This study extends these findings by demonstrating how embodied musical engagement fosters both short-term emotional regulation and long-term cognitive resilience, suggesting a need for further longitudinal research on sustained impact.

All themes illustrate embodied engagement in a concrete context and are also strikingly in concurrence with the concept of 4E cognition (Schiavio and van der Schyff, 2018), i.e., embodied, embedded, enacted, and extended cognition. Activities such as rhythmic singing and vocal improvisation simultaneously activated embodied, embedded, enacted, and extended cognitive processes: The sensory, situated, and highly interactive practices, aided by devices such as infographics and images, thus align with supporting children’s ability to navigate trauma and enhance their sense of belonging. This holistic integration corroborates previous studies emphasizing the role of physicality in emotional regulation and wellbeing (Nijs and Nicolaou, 2021; Juntunen and Sutela, 2023). Hence, the findings indicate that adopting an inclusive perspective on designing learning environments for refugee children may justifiably involve a shift in the perception of facilitators’ professional roles, as Schmid (2024, p. 12) has stated. Human flourishing can be a musical objective, while singing may apparently become an act of care (Holdhus, 2023, p. 421): the research highlights how singing practices that are contextualized in a multimodal, specific learning environment, intersect with the principles of caring thinking, as introduced by Lipman (2003). Evidently, singing-based activities, particularly those involving collaborative vocal improvisation and storytelling, were centered around empathy and ethical reflection. Children engaged in shared narratives that promoted emotional attunement, thereby fostering relational understanding and empathy core elements of caring thinking. In total, the study underscores the relevance of the dimension of embodiment but also of narrativity, materiality, and sociality in addition to the dimensions of play, multimodality, spatiality, and agency as Oravec and Schmid (2024) have elaborated with respect to the fostering of an equitable musical-aesthetic practice for primary-school-aged children in general. However, educational settings must be considerate of refugee children’s special needs.

### 5.2 Implications for educational practice with refugee children

The results offer several practical insights for developing singing-based educational interventions for refugee children:

1. Embedding Singing-Based Embodied Practices in Curriculum: Incorporating singing as a regular component of education provides a non-verbal platform for refugee children to process trauma and develop emotional regulation skills. Singing can facilitate both mindfulness and self-expression through structured activities like vocal improvisation and rhythmic breathing exercises. These embodied experiences offer an effective complement to traditional academic learning, addressing socio-emotional needs in a trauma-informed manner (Woodard, 2024).
2. Culturally responsive pedagogies: Singing can serve as a powerful tool for bridging cultural and linguistic divides. This study found that children eagerly shared and adopted each other’s traditional songs, creating opportunities for cross-cultural dialogue and mutual respect. By integrating culturally relevant singing practices into educational programs, educators can promote a sense of identity and belonging while fostering inclusive classroom environments. Research by Nicolaou et al. (2023) similarly emphasizes the importance of participatory singing in asylum centers, where it supports both cultural expression and social bonding.
3. Benefits of singing: Singing, particularly in group settings, provides a structured and safe outlet for emotional expression. Synchronized vocal activities such as choral singing and vocal play can help reduce performance anxiety and foster trust among participants. Juntunen and Sutela (2023) demonstrate that these embodied practices enhance resilience by promoting active engagement and self-regulation. Refugee children benefit from these interventions by developing long-term coping strategies to manage stress and trauma. As a matter of fact, the facilitator’s role is not a therapeutic one, as Schmid (2024) emphasizes. Nevertheless, secondary effects such as the ones described above seem to be inherent in the discipline and may be reclaimed as primary effects (p. 13).
4. Facilitator training for singing-based interventions: The role of the facilitator is crucial in creating supportive, embodied learning environments that encourage children’s participation and emotional exploration. Nicolaou et al. (2023) emphasize the importance of reflexivity in facilitator practice, noting that artist-facilitators must continuously adapt singing activities to meet participants’ evolving emotional and cultural needs. This aligns with de Quadros and Amrein (2023) concept of *empowering song*, which repositions the facilitator as a co-musicker who engages in culturally responsive, justice-driven musical practice rather than acting as a fixed authority figure. Facilitators should be trained to model embodied engagement [e.g., consciously utilizing the role of gestures (p. 8)] and guide children through emotionally sensitive activities, thereby fostering trust, empathy, and collaboration.

### 5.3 Limitations

This study has several limitations. First, the small sample size, single-site design, wide age range (7–12), and culturally heterogeneous cohort limit the generalizability of the findings. Second, the intervention dosage was brief (approximately 2 h per week) and our analyses focused on short-term processes; longitudinal research is needed to examine sustained effects on socio-emotional learning. Third, because singing, vocal expression, storytelling, and movement were implemented concurrently as an integrated design, disentangling the specific contribution of each component was not possible; future studies should isolate or systematically vary components to enable clearer attribution. Fourth, replication in mainstream classrooms of around 25 students may be constrained by heterogeneous needs and logistical factors; adapting core DBR features (e.g., continuous feedback, adaptive facilitation, student participation) to small-group routines could improve feasibility but require testing at larger scale. Finally, comparative studies with non-refugee children would help determine which effects are context-specific versus more general.

## 6 Conclusion

This study underscores the transformative potential of embodied musical practices in fostering emotional resilience, social integration, and cognitive engagement among refugee children. By designing and implementing the Multimodal Singing-Based Learning Environment (MSLE), this research provided valuable insights into how embodied learning can address the holistic needs of vulnerable populations. The findings revealed that embodied practices, such as rhythmic singing and storytelling, enable children to process emotions, build empathy, and strengthen their sense of belonging. Grounded in the theoretical framework of 4E cognition, embodied, embedded, enacted, and extended this study demonstrated the interconnectedness of mind, body, and emotions in creating inclusive and responsive educational settings. Furthermore, the integration of caring thinking within these practices fostered collaborative exploration of emotions and values, providing a structured approach for children to navigate the social and emotional complexities of displacement.

The study’s contributions extend beyond the immediate context of refugee education. By embedding cultural elements, such as traditional songs and narratives, into learning environments, the research highlights the importance of culturally responsive pedagogy in empowering children to reconnect with their identities while fostering mutual respect in diverse groups. The iterative and participant-centered approach of the Design-Based Research (DBR) framework offers a replicable model for educators seeking to develop adaptable, trauma-informed interventions in comparable contexts such as refugee camps.

In conclusion, this study highlights the potential of embodiment in multimodal learning environments, addressing the needs of refugee children. By bridging theoretical insights with practical applications, it offers a framework for creating inclusive and supportive educational settings. Moreover, the role of the facilitator was shown to be central in ensuring that such inclusive environments are not only designed but lived through active, relational pedagogy. Recognizing facilitators as co-learners and cultural mediators is thus essential for sustaining the emotional and intercultural integrity of embodied, music-based learning environments. Future research should continue to refine and expand these approaches, paving the way for innovative interventions that empower refugee children to thrive emotionally, socially, and academically in diverse contexts.

## Data Availability

Due to ethical and privacy restrictions of refugee children, the raw video data collected for this study cannot be shared publicly. Only anonymized/aggregated data (or [specify: transcripts, coded data, etc.]) supporting the findings are available from the corresponding author on reasonable request.

## Footnotes

^1^It must be stated that given the participatory setting and data collection approach (e.g., Mosaic approach), the research is currently more and more leaning towards a Participatory Action Research Approach (PAR) which will influence the second research cycle Banffy-Hall (2015). However, the data analysed in this paper was collected during the first (DBR) cycle.

^2^https://www.instagram.com/mahsa.m.hoseiny/.

^3^ https://youtu.be/v1k4w6GJgTo?si=mG6czq9vshAkCJBq

^4^In general, it is also important to be mindful of the term *“refugee children,”* as the focus on this group can be subject to debate. Ideally, equity-based approaches follow an inclusive, non-segregating philosophy. However, the existence of targeted programs for these children—particularly in contexts such as refugee camps—creates a need for empirically grounded concepts.
